# Factors associated with adverse outcomes from cardiovascular events in the kidney transplant population: an analysis of national discharge data, hospital characteristics, and process measures

**DOI:** 10.1186/s12882-019-1390-2

**Published:** 2019-05-28

**Authors:** Amit K. Mathur, Yu-Hui Chang, D. Eric Steidley, Raymond L. Heilman, Nabil Wasif, David Etzioni, Kunam S. Reddy, Adyr A. Moss

**Affiliations:** 1 Division of Transplant Surgery, Department of Surgeyr, Mayo Clinic Arizona, 5777 East Mayo Boulevard, Phoenix, AZ 85054 USA; 2 Robert D. and Patricia E. Kern Center for the Science of Health Care Delivery, Mayo Clinic, Phoenix, AZ USA; 3 Division of Cardiovascular Medicine, Mayo Clinic Arizona, Phoenix, AZ USA; 4 Division of Nephrology, Mayo Clinic Arizona, Phoenix, AZ USA

**Keywords:** Cardiovascular disease, Care delivery, Economics, Kidney transplant

## Abstract

**Background:**

Kidney transplant (KT) patients presenting with cardiovascular (CVD) events are being managed increasingly in non-transplant facilities. We aimed to identify drivers of mortality and costs, including transplant hospital status.

**Methods:**

Data from the 2009–2011 Nationwide Inpatient Sample, the American Hospital Association, and Hospital Compare were used to evaluate post-KT patients hospitalized for MI, CHF, stroke, cardiac arrest, dysrhythmia, and malignant hypertension. We used generalized estimating equations to identify clinical, structural, and process factors associated with risk-adjusted mortality and high cost hospitalization (HCH).

**Results:**

Data on 7803 admissions were abstracted from 275 hospitals. Transplant hospitals had lower crude mortality (3.0% vs. 3.8%, *p* = 0.06), and higher un-adjusted total episodic costs (Median $33,271 vs. $28,022, *p* < 0.0001). After risk-adjusting for clinical, structural, and process factors, mortality predictors included: age, CVD burden, CV destination hospital, diagnostic cardiac catheterization without intervention (all, *p* < 0.001). Female sex, race, documented co-morbidities, and hospital teaching status were protective (all, *p* < 0.05). Transplant and non-transplant hospitals had similar risk-adjusted mortality. HCH was associated with: age, CVD burden, CV procedures, and staffing patterns. Hospitalizations at transplant facilities had 37% lower risk-adjusted odds of HCH. Cardiovascular process measures were not associated with adverse outcomes.

**Conclusion:**

KT patients presenting with CVD events had similar risk-adjusted mortality at transplant and non-transplant hospitals, but high cost care was less likely in transplant hospitals. Transplant hospitals may provide better value in cardiovascular care for transplant patients. These data have significant implications for patients, transplant and non-transplant providers, and payers.

## Background

Cardiovascular events are the leading cause of death after kidney transplantation (KT). Significant amounts of research have been aimed at reducing event rates, primarily aimed at understanding prevalent risk factors, defining outcomes, and application of guideline-based care [1–4]. Event rates continue to be high and endanger long-term patient and transplant outcomes.

Post-KT cardiovascular event are among the most important drivers of post-kidney transplant health care utilization and mortality [5]. KT recipients have high rates of hospitalization for myocardial infarction (MI), congestive heart failure (CHF), dysrhythmias, stroke (CVA), malignant hypertension, and cardiac arrest. Mortality is as high as to 20% in some hospitals. Few studies have focused on the rescue of KT patients once these events occur [6]. Patient and hospital factors may contribute to adverse outcomes from CVD events. Hospitals are known to vary in cardiac care practices [7–11], and structural features including teaching status, technology, and staffing patterns are associated with better outcomes [12]. KT patients bring an even greater challenge in this setting – rescue from an acute cardiovascular event requires facility resources and well-developed care processes, which can be leveraged from transplant programs. The presence of these resources may improve outcomes and reduce costs of cardiovascular care, but this idea remains unexplored.

In this analysis, we aimed to understand how hospitals perform in the management of cardiovascular disease in kidney transplant patients. We modeled hospital characteristics including structural factors and cardiovascular process measures as well as clinical factors to identify predictors of inpatient mortality and costs [13]. We hypothesized that transplant hospitals (TH) would have lower mortality and costs compared to non-transplant hospitals (NTH), after adjustment for their inherent characteristics and patient differences.

## Methods

### Conceptual model

**Fig. 1:**
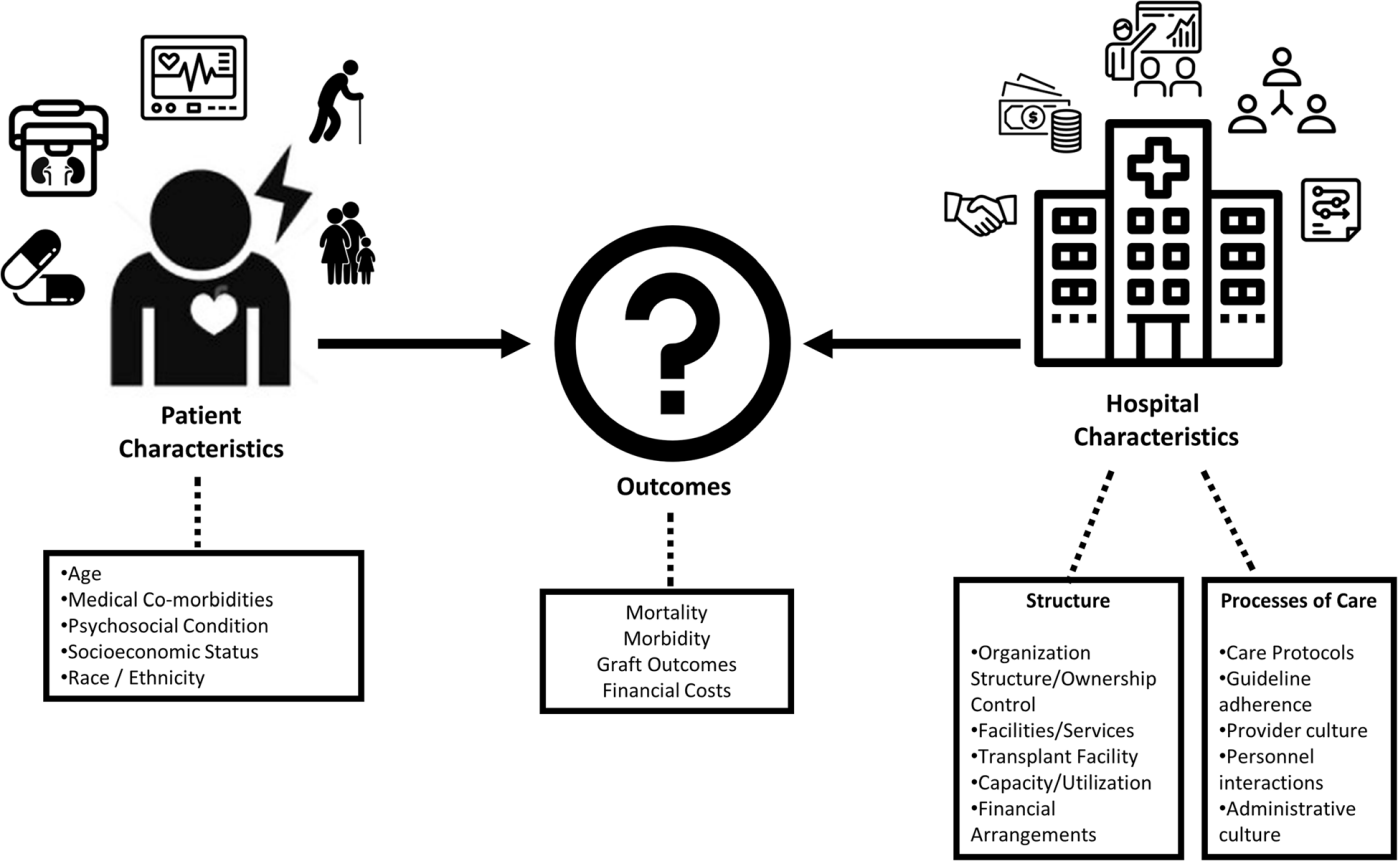
Drivers of Clinical and Financial Outcomes for Kidney Transplant Patients Admitted for Inpatient Care for Cardiovascular Events. This conceptual model encapsulates the factors that contribute to outcomes when a kidney transplant recipient is admitted for inpatient care for a cardiovascular event. Kidney transplant patients admitted to hospitals with cardiovascular dysfunction are a complex population. They require multi-disciplinary coordinated care, multiple levels of acute inpatient care, comprehensive nephrology and cardiology services, specialized imaging and therapeutic capabilities, and other resources to address deterioration of kidney function, threatened allografts, immunosuppression, and numerous other queries. Well-resourced hospitals may be better equipped to prevent adverse outcomes when cardiovascular events occur in this population. Our analysis employed multi-level statistical models based on the Donabedian model of health care quality, with risk-adjustment for 1) patient characteristics, 2) key facility structural factors, which include organizational elements, facilities and services, as well as ownership and financial status, and 3) processes of care that appropriately utilize facilities and services to deliver treatment. Based on this structure, we hypothesized that transplant hospitals would have better clinical and financial outcomes than non-transplant hospitals from cardiovascular events in the kidney transplant population

Figure 1 displays a conceptual model of factors that affect outcomes when kidney transplant recipients have cardiovascular events. We considered patient-level and hospital-level factors (structure and processes of care) that could affect outcomes in this population. In this context, resource intensity - the presence of specialty cardiac services, intensive care, teaching status, nurse staffing and other factors - would be associated with favorable outcomes, after adjusting for patient differences.

### Data sources

Using data from the Nationwide Inpatient Sample (NIS), the American Hospital Associatation (AHA) Annual Survey of Hospitals, and Hospital Compare we created a novel dataset capturing admissions from kidney transplant patients admitted with cardiovascular events based on specific diagnoses, merged with hospital resource characteristics and cardiovascular process measures from 2009 to 2011, as previously described [6, 14]. The Nationwide Inpatient Sample (NIS) is a 20% de-identified national administrative data sample of all U.S. hospital discharges which contains hospital episode-based patient demographics, clinical diagnoses and treatments based on 9th International Classification of Disease (ICD-9) codes. The American Hospital Association (AHA) Annual Survey of Hospitals provided hospital structural characteristics using the Medicare provider number. Survey data includes 1000 data elements on organizational structure, facilities, payer mix, and financial performance from 6500 U.S. hospitals. Structural domains included in the model included TH status, hospital finances, inpatient and cardiovascular care capacity, staffing patterns and teaching status. Cardiovascular process metrics and outcomes are published by the Centers for Medicare and Medicaid Services on the Hospital Compare website (http://www.medicare.gov/hospitalcompare), and were merged by Medicare provider number. The metrics used included time to ECG on arrival, incidence of aspirin on arrival to ED for MI, proportion of MI patients receiving fibrinolytic therapy within 30 min, and time to transfer to another facility for acute coronary intervention. Hospital outcome metrics included baseline rates of inpatient 30-day mortality and readmission for MI and CHF.

To create the final study population, kidney transplant patients (V42.0, kidney transplant status) who were admitted with at least one primary or secondary cardiovascular diagnosis were isolated. Cardiovascular diagnoses included: myocardial infarction (MI) (410.x), congestive heart failure (CHF) (428.x), dysrhythmia (427.x), cerebrovascular accident (CVA) (436.x, 437.1, 997.x), malignant hypertension (402.x), and cardiac arrest (427.5, 997.1). Multi-organ transplants were omitted. The final models were limited to patients with functioning allografts by restricting the dataset to those records without billing codes for inpatient dialysis use (14.2%), which are most relevant to transplant quality metrics. The final dataset was restricted to hospitals with greater than 10 admissions. Clinical risk-adjustment was based on the presence diabetes mellitus and the Charlson comorbidity score. Hospital cost-to-charge ratios provided by the Centers for Medicare and Medicaid Services were used to to determine episodic costs, as described previously [15]. The GDP Implicit Price Deflator was used to adjust for inflation, centered on 2011 dollars [16].

Patient socio-demographics and clinical data, facility structural characteristics, process measures, and baseline hospital cardiovascular performance metrics (in non-transplant patients) used in final models are displayed in Table 1. Facility characteristics such as total hospital expenses (which are expressed in the AHA dataset in US dollars) and total inpatient days, were ranked and divided into quartiles for presentation.

**Table 1:** Differences in Demographic, Clinical, and Facility Characteristics among Kidney Transplant Patients Admitted with Cardiovascular Disease, by Transplant Hospital Status

Demographic and Clinical Characteristics				
	Non-transplant Hospital (*n* = 197)	Transplant Hospital (*n* = 78)	Total (*n* = 275)	*p*-value
Hospitalizations (n,%)	3893, 49.9%	3910, 50.1%		
Year of admission				< 0.001
2009	1182 (30.4%)	1349 (34.5%)	2531 (32.4%)	
2010	1238 (31.8%)	1155 (29.5%)	2393 (30.7%)	
2011	1473 (37.8%)	1406 (36.0%)	2879 (36.9%)	
Age, Median (Q1, Q3)	64 (55.0, 70.0)	62 (53.0, 69.0)	63 (54.0, 70.0)	< 0.001
Female	1484 (38.1%)	1453 (37.2%)	2937 (37.6%)	0.37
Race, White	2337 (64.4%)	2109 (57.4%)	4446 (60.9%)	< 0.001
Cardiovascular Diagnosis				
MI (410.x)	383 (9.8%)	305 (7.8%)	688 (8.8%)	0.002
Stroke (997.x/436/437.1)	247 (6.3%)	461 (11.8%)	708 (9.1%)	< 0.001
CHF (428.x)	2110 (54.2%)	1968 (50.3%)	4078 (52.3%)	< 0.001
Dysrhythmia (427.x)	2138 (54.9%)	2027 (51.8%)	4165 (53.4%)	0.006
Cardiac arrest (427.5/997.1)	85 (2.2%)	102 (2.6%)	187 (2.4%)	0.22
Malignant HTN (402.x)	59 (1.5%)	85 (2.2%)	144 (1.8%)	0.03
Number of CV diagnosis				0.02
1	2902 (74.5%)	3005 (76.9%)	5907 (75.7%)	
≥ 2	991 (25.5%)	905 (23.1%)	1896 (24.3%)	
Weighted Charlson score				< 0.001
0	467 (12.0%)	828 (21.2%)	1295 (16.6%)	
1	471 (12.1%)	754 (19.3%)	1225 (15.7%)	
2	1158 (29.7%)	1102 (28.2%)	2260 (29.0%)	
3+	1797 (46.2%)	1226 (31.4%)	3023 (38.7%)	
Diabetes mellitus	2012 (51.7%)	1959 (50.1%)	3971 (50.9%)	0.16
Dialysis use in hospital	749 (19.2%)	356 (9.1%)	1105 (14.2%)	< 0.001
Admission type				< 0.001
Emergent/Urgent	2884 (88.3%)	2850 (83.1%)	5734 (85.6%)	
Elective/Others	383 (11.7%)	581 (16.9%)	964 (14.4%)	
Transferred in indicator	302 (7.8%)	509 (13.0%)	811 (10.4%)	< 0.001
Cardiovascular Procedure (catheter-based or cardiac surgery)	757 (19.5%)	772 (19.7%)	1529 (19.6%)	0.74
Died in hospital	146 (3.8%)	117 (3.0%)	263 (3.4%)	0.06
Facility Structural Characteristics				
Hospital Type				0.09
Government, nonfederal	14 (7.1%)	12 (15.4%)	26 (9.5%)	
Non-profit, non-gov’t	175 (88.8%)	62 (79.5%)	237 (86.2%)	
Investor-owned	8 (4.1%)	4 (5.1%)	12 (4.4%)	
Medical/surgical intensive care	191 (97.0%)	78 (100.0%)	269 (97.8%)	0.12
Cardiac intensive care	141 (71.6%)	71 (91.0%)	212 (77.1%)	< 0.001
HMO hospital	17 (8.6%)	9 (11.5%)	26 (9.5%)	0.46
PPO hospital	16 (8.1%)	3 (3.8%)	19 (6.9%)	0.21
Specialty cardiology & cardiac surgery services	186 (94.4%)	77 (98.7%)	263 (95.6%)	0.12
Freestanding/Satellite ED hospital	25 (12.7%)	6 (7.7%)	31 (11.3%)	0.24
Multi-detector 64-slice spiral CT	171 (86.8%)	75 (96.2%)	246 (89.5%)	0.02
Radiology interventional therapy	131 (66.5%)	73 (93.6%)	204 (74.2%)	< 0.001
Hospital unit inpatient days				< 0.001
First quartile	62 (31.5%)	6 (7.7%)	68 (24.7%)	
Second quartile	58 (29.4%)	11 (14.1%)	69 (25.1%)	
Third quartile	51 (25.9%)	18 (23.1%)	69 (25.1%)	
Fourth quartile	26 (13.2%)	43 (55.1%)	69 (25.1%)	
Proportion of hospital unit Medicare discharges				< 0.001
Median (Q1, Q3)	0.5 (0.4, 0.5)	0.4 (0.3, 0.4)	0.4 (0.4, 0.5)	
Proportion of hospital unit Medicaid discharges				< 0.001
Median (Q1, Q3)	0.1 (0.1, 0.2)	0.2 (0.2, 0.3)	0.2 (0.1, 0.2)	
Number of Operating Rooms				< 0.001
Median (Q1, Q3)	14 (10.0, 18.0)	26 (19.0, 37.0)	16 (11.0, 24.0)	
Total surgical operations				< 0.001
Median (Q1, Q3)	10,927	20,764	12,152	
	(8018, 15,101)	(11,209, 27,502)	(8504, 19,382)	
Surgical intensity (Surgical procedures/inpatient beds/year)				0.31
Median (Q1, Q3)	35.3	37.8	36.5	
	(26.6, 46.5)	(30.1, 48.4)	(27.6, 47.1)	
Hospital total expenses				< 0.001
First quartile	62 (31.5%)	6 (7.7%)	68 (24.7%)	
Second quartile	60 (30.5%)	9 (11.5%)	69 (25.1%)	
Third quartile	58 (29.4%)	11 (14.1%)	69 (25.1%)	
Fourth quartile	17 (8.6%)	52 (66.7%)	69 (25.1%)	
Physician FTEs/10 beds				0.62
Median	0.3	0.3	0.3	
Q1, Q3	0, 1.1	0, 1.7	0, 1.2	
Range	(0–23.5)	(0–24.6)	(0–24.6)	
Teaching Status				< 0.001
Nonteaching	90 (54.2%)	18 (24.7%)	108 (45.2%)	
Minor teaching	61 (36.8%)	15 (20.5%)	76 (20.6%)	
Major teaching	15 (9.0%)	40 (54.8%)	55 (54.8%)	
FTEs nurses/10 beds				< 0.001
Median	16.8	22.2	17.6	
Q1, Q3	13.8, 19.9	17.4, 26.9	14.3, 22.3	
Range	(0.2–47.3)	(0.2–41.6)	(0.2–47.3)	
Hospital Process Factors – Timely & Effective Care: Heart Attack				
Fibrinolytic therapy received within 30 min of ED arrival (percentage)	*N* = 58	*N* = 24	*N* = 82	0.88
Median	50	41.5	50	
Q1, Q3	0, 100	0, 100	0, 100	
Aspirin at arrival (percentage)	*N* = 194	*N* = 77	*N* = 271	0.18
Median	100	100	100	
Q1, Q3	99, 100	99, 100	99, 100	
Time to transfer to another facility for acute coronary intervention (minutes)	*N* = 37	*N* = 7	*N* = 44	0.08
Median	78	42	73.5	
Q1, Q3	57, 105	30, 121	51, 106.5	
Time to ECG (minutes)	*N* = 161	*N* = 51	*N* = 212	0.01
Median	11	16	12	
Q1, Q3	8, 18	8, 26	8, 19	
Hospital Mortality and Unplanned Hospital Visits				
Acute myocardial infarction 30-day mortality rate (percentage)	*N* = 183	*N* = 75	*N* = 258	0.31
Median	16.1	15.9	16	
Q1, Q3	15.1, 17.1	14.6, 17.4	14.9, 17.1	
Heart failure 30-day mortality rate (percentage)	*N* = 186	*N* = 76	*N* = 262	0.10
Median	10.9	10.7	10.8	
Q1, Q3	10, 12	9.8, 11.8	10, 12	
Acute myocardial infarction 30-day readmission rate (percentage)	*N* = 183	*N* = 76	*N* = 259	0.13
Median	20.1	20.5	20.2	
Q1, Q3	19.2, 21.4	19.7, 21.5	19.3, 21.4	
Heart failure 30-day readmission rate (percentage)	*N* = 186	*N* = 76	*N* = 262	0.83
Median	24.7	25.0	24.8	
Q1, Q3	23.4, 26.6	23.4, 26.8	23.4, 26.7	

### Statistical analysis

#### Determinants of mortality

We constructed generalized estimating equations (GEE) to identify factors associated with mortality [17], while accounting for patient clustering by hospital, as individual hospitals possess unique structural and process characteristics that could affect all patients within their cluster. Structural and process of care variables were included to address clustering. The Classification and Regression Tree (CART) method to identify relevant hospital variables associated with mortality and hospital transplant status for multivariate analysis [18]. The CART method optimizes the classification of observations into mutually exclusive groups in a non-parametric approach. The method identifies a single variable able to strongly divide observations into two groups. The observations are further sub-divided within groups using the same method in an iterative process, until pre-specified stopping rules are met.

GEE estimates were used to construct the odds ratio (OR) and the 95% CI for individual covariates, after applying backward elimination techniques to select the best model (retained variables had *p*-value < 0.4). The quasi-likelihood under independence model criterion (QIC) was used to measure model fitness, and compared across three models: the model with transplant status only, the model with transplant status and patient characteristics, and the model with transplant status, patient and hospital characteristics [19]. Similar to the Akaike’s information criterion, lower values indicate better model fit. We expected to observe the highest QIC from the empty model with transplant status only, and the lowest QIC from the model with both patient and hospital characteristics.

#### Determinants of high cost cardiovascular hospitalizations

Hospitalizations were grouped into cost quartiles after conversion of hospitalization charges. We utilized the CART method and similarly structured GEEs as described above for mortality to determine the predictors of high cost hospitalization (highest cost quartile). The backward elimination technique was used to select the best model, and the variables with *p*-value < 0.4 were retained in the model, and model fitness was assessed using QIC.

The project was exempt from IRB approval as the data utilized were publicly available and de-identified. The analysis was conducted in SAS 9.4 (SAS Institute) and in R 3.1.3 (R Foundation for Statistical Computing). All tests were two-sided, and a *p*-value < 0.05 was considered statistically significant.

## Results

The final analysis sample consisted of 7803 hospital admissions from 275 hospitals from 2009 to 2011. Among the 275 hospitals, 28% (*n* = 78) were kidney transplant facilities and 72% (*n* = 197) were non-transplant facilities. Cardiovascular hospitalizations in the KT population were evenly distributed between NTHs (*n* = 3893, 49.8%) and THs (*n* = 3910, 50.1%). CHF and dysrhythmia were the leading causes of admission. The descriptive statistics for patient and hospital characteristics are shown in Table 1.

There were significant population differences in THs versus NTHs (Table 1). NTHs had more white patients. Multiple CVDs were coded in 24.3% of cases, more often at NTHs. NTHs had a significantly higher proportion of MI, CHF, and dysrhythmia admissions, while THs had more stroke, cardiac arrest, and malignant hypertension admissions. NTHs admitted significantly more patients with a high co-morbidity burden by Charlson score. Diabetes mellitus was commonly coded in hospitalizations at both types of facilities, and was present in the majority of admissions (> 50%). NTHs had a significantly greater proportion of emergent/urgent admissions. NTHs demonstrated longer lengths of stay versus THs.

Facility characteristics differed between TH and NTHs. Cardiac intensive care was significantly more prevalent in THs vs. NTHs, but there was similar prevalence of specialty cardiac services in both hospital types. THs had significantly more surgical volume and daily occupancy. THs had significantly more technology (presence of 64-slice CT scanners and interventional radiology therapy). Case-mix was significantly different between the two hospital types, with THs had more Medicaid patients and NTHs had more Medicare. THs had higher total expenses, staffing ratios, and major teaching efforts compared to their counterparts.

Four cardiovascular process of care measures were available for analysis: time to receipt of fibrinolytic therapy, time to receipt of electrocardiogram, receipt of aspirin on hospital admission, and time to transfer to another facility for acute coronary intervention. THs and NTHs were similar in these, but these were not fully reported during the study period.

Table 2 demonstrates differences in mortality and proportion of high cost admissions stratified by primary cardiovascular diagnosis. THs and NTHs had similar rates of mortality and high cost admissions in MI, stroke, and dysrhythmia. Mortality from CHF was significantly higher in NTHs compared to THs, but had a similar proportion of high cost admissions. For patients admitted in cardiac arrest, mortality was similar, between 31 to 34%, but 73.7% of those admissions were high cost in THs compared to 57.3% in NTHs.

**Table 2:** Variation in mortality and high cost admissions by diagnosis and transplant hospital status

Primary CV diagnosis	Mortality			High cost (in the top quartile)		
	Non-transplant hospital	Transplant hospital	*p*-value	Non-transplant hospital	Transplant hospital	*p*-value
MI (410.x)	7.0%	7.9%	0.68	44.3%	51.2%	0.10
	(27/383)	(24/305)		(143/323)	(125/244)	
Stroke (997.x/436/437.1)	5.7%	3.5%	0.17	41.1%	45.1%	0.34
	(14/247)	(16/461)		(85/207)	(185/410)	
CHF (428.x)	3.4%	2.3%	0.04	23.1%	24.1%	0.48
	(72/2110)	(46/1968)		(417/1807)	(389/1614)	
Dysrhythmia (427.x)	4.7%	4.0%	0.25	21.5%	27.3%	< 0.001
	(101/2138)	(81/2027)		(395/1838)	(466/1704)	
Cardiac arrest (427.5/997.1)	34.1%	31.4%	0.69	57.3%	73.5%	0.03
	(29/85)	(32/102)		(43/75)	(61/83)	
Malignant HTN (402.x)	0	0		9.3%	13.2%	0.49
				(5/54)	(10–76)	

### Hospital-specific mortality: crude and risk-adjusted analyses

**Fig. 2:**
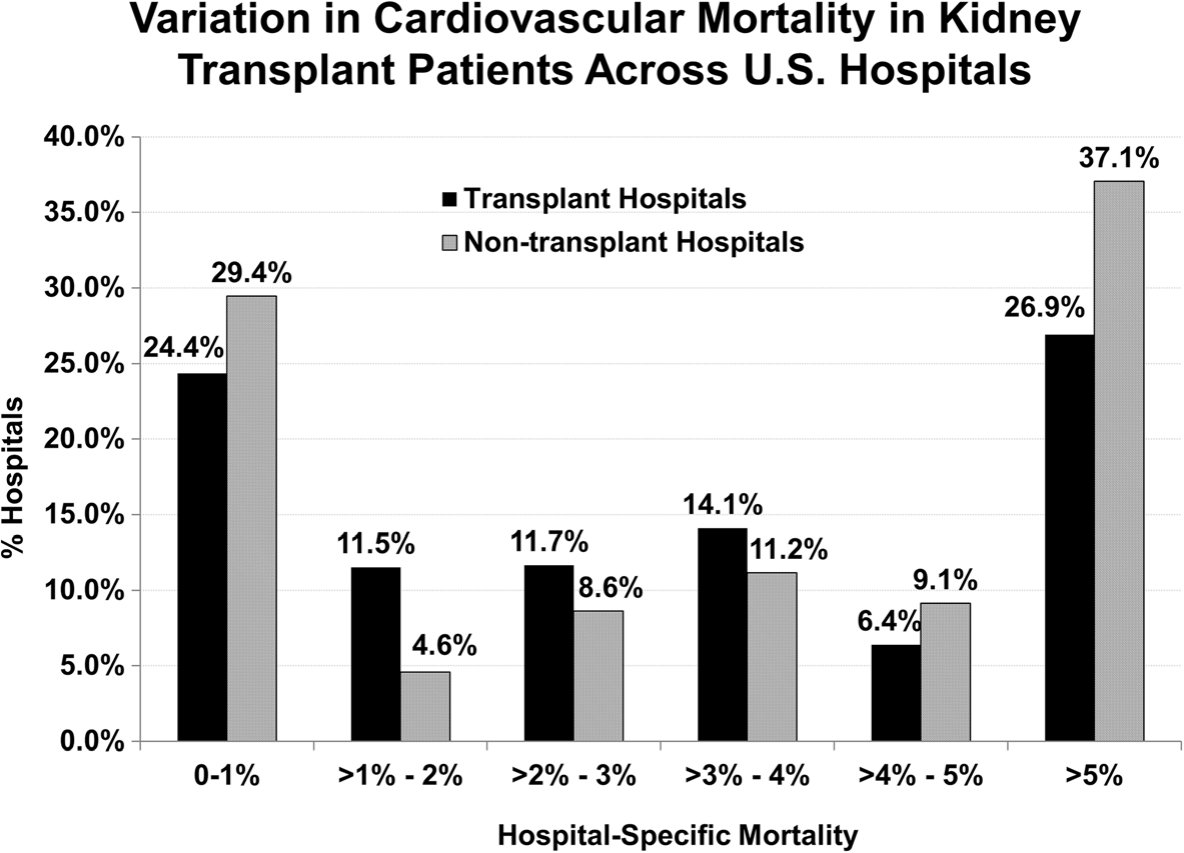
Variation in cardiovascular mortality in kidney transplant patients across U.S. hospitals. Across a broad sample of hospitals in the United States, among kidney transplant recipients, there was tremendous variation noted in un-adjusted mortality for patients admitted with a cardiovascular disease process (including myocardial infarction, stroke, congestive heart failure, dysrhythmia, cardiac arrest, or malignant hypertension). The overall hospital mortality was 3.4%, but had a wide range. The crude mortality trends indicated that non-transplant hospitals were over-represented in both low and high mortality outlier groups. A greater proportion of non-transplant hospitals (37.1%) were designated as high mortality hospitals (> 5%) compared to transplant hospitals (26.9%). This relationship was not uniform across all hospital-specific mortality categories, which warranted further analysis using multivariate hierarchical models

Overall in-hospital cardiovascular mortality was 3.4% (263/7802, with one admission with missing mortality information), and trended toward higher rates in NTHs versus THs (3.8% vs. 3.0%, *p* = 0.06). Figure 2 demonstrates variation in the distribution of hospital-specific cardiovascular mortality across all hospitals. Median hospital-specific mortality was 3.4%, but varied significantly across hospitals (IQR 0–5.9%, range 0–21%). Hospital-specific mortality had a bimodal distribution, with more than a third of hospitals demonstrating more than 5% cardiovascular mortality in KT recipients. Among higher mortality hospitals, there was a higher proportion of NTHs.

On multivariable analysis, we identified several clinical and hospital characteristics associated with mortality (Table 3). Importantly, sequential addition of patient and then hospital characteristics improved model performance by QIC. TH status was not associated with mortality, even after including patient and hospital characteristics. Multiple clinical characteristics were important drivers of mortality. Age, burden of cardiovascular diagnoses, and utilization of diagnostic cardiac catheterization (but not therapeutic catheterization) were associated with significantly higher mortality, respectively. Female sex, non-white race, and documentation of co-morbidities (diabetes mellitus and dyslipidemia) were associated with lower mortality, respectively. Admission to a cardiovascular destination hospital (high proportion of cohort patients transferred in this facility), and hospitals with long lengths of stay were factors associated with mortality. The only hospital factor that was protective from mortality was major teaching status, which reduced the odds of mortality by 68% compared to non-teaching facilities (OR 0.32, *p* = 0.002). Cardiovascular process of care measures including time to ECG and ASA administration at arrival were not significant predictors of mortality.

**Table 3:** Characteristics Associated with Inpatient Mortality from Cardiovascular Disease after Kidney Transplantation

Variable	Comparison	Model 1: Transplant Hospital Only				Model 2: Transplant Hospital + Patient Characteristics				Model 3: Transplant Hospital + Patient Characteristics + Hospital Characteristics			
		OR	95% CI		*p*-value	OR	95% CI		*p*-value	OR	95% CI		*p*-value
Transplant hospital	Transplant vs. Non-transplant	0.81	0.60	1.09	0.16	0.72	0.48	1.08	0.11	0.98	0.50	1.91	0.94
Patient-level Characteristics													
Age	≥60 vs. < 60					2.28	1.53	3.4	< 0.001	2.29	1.37	3.85	0.002
Race	Non-white vs. White					0.62	0.39	0.99	0.04	0.49	0.26	0.9	0.02
Sex	Female vs. Male					0.69	0.47	1.02	0.06	0.59	0.37	0.92	0.02
Type of admission	Emergent/Urgent vs. Elective/Others					1.37	0.73	2.56	0.33	1.5	0.69	3.26	0.30
Admitted to high transfer in hospital	Yes vs. No					1.52	1.02	2.27	0.04	1.76	1.01	3.05	0.05
Number of CV diagnosis	≥2 vs. 1					2.09	1.44	3.03	< 0.001	1.73	1.08	2.78	0.02
Weighted Charlson score	≥2 vs. 0–1					1.37	0.89	2.12	0.16	1.06	0.63	1.81	0.82
Hypertension	Yes vs. No					1.11	0.64	1.93	0.71	1.26	0.61	2.57	0.53
Tobacco abuse	Yes vs. No					0.39	0.09	1.68	0.21	0.24	0.03	2.05	0.19
Dyslipidemia	Yes vs. No					0.48	0.31	0.75	0.001	0.58	0.35	0.98	0.04
Diabetes mellitus	Yes vs. No					0.46	0.31	0.66	< 0.001	0.4	0.25	0.64	< 0.001
Invasive CV procedure													
Diagnostic Cardiac catheterization	Yes vs. No					2.60	1.76	3.84	< 0.001	2.1	1.32	3.36	0.002
Therapeutic cardiac catheterization	Yes vs. No					0.22	0.07	0.67	0.008	0.35	0.1	1.17	0.09
CABG	Yes vs. No					0.23	0.06	0.93	0.04	0.35	0.07	1.65	0.18
Valve surgery	Yes vs. No					0.51	0.12	2.12	0.35	0.38	0.07	1.95	0.25
Other cardiac surgery	Yes vs. No					2.94	0.79	11.01	0.11	1.26	0.2	7.88	0.81
Hospital-level Characteristics													
Owner, Financial status, Payer Mix													
Hospital total expenses by quartile	2 vs. 1									1.45	0.64	3.27	0.38
	3 vs. 1									0.52	0.16	1.68	0.28
	4 vs. 1									0.26	0.07	0.97	0.05
Inpatient Capacity													
Hospital unit inpatient days by quartile	2 vs. 1									0.53	0.21	1.3	0.16
	3 vs. 1									1.02	0.34	3.11	0.97
	4 vs. 1									4.26	1.2	15.21	0.03
Cardiac intensive care	Yes vs. No									1.75	0.78	3.91	0.18
Staffing Patterns													
Teaching status	Minor teaching vs. Nonteaching									0.65	0.37	1.15	0.14
	Major teaching vs. Nonteaching									0.32	0.16	0.65	0.002
Process of Care for Heart Attack													
Aspirin at arrival	Per 5% increase									2.28	0.58	9.07	0.24
Time to ECG	Per 20 min increase									0.98	0.81	1.19	0.85
QIC		1758.36				1095.68				736.47			

### Hospital-specific hospitalization cost: crude and risk-adjusted analyses

**Fig. 3:**
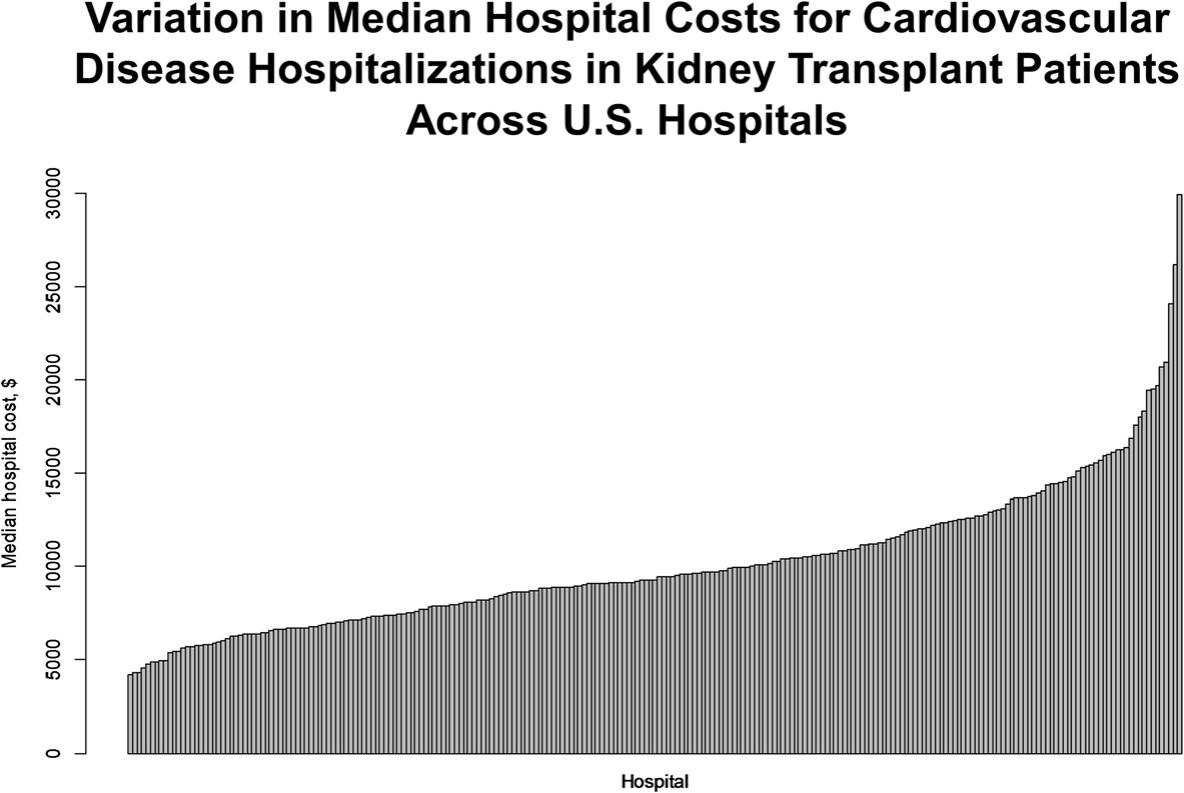
Variation in median hospital costs for cardiovascular disease hospitalizations in kidney transplant patients across U.S. hospitals. Similar to variation in hospital mortality, we observed vast differences in median hospital costs for inpatient care for cardiovascular disease among kidney transplant recipients. Each bar represents an individual hospital in the analysis, and the degree of median cost-variation varied nearly six-fold

Figure 3 demonstrates the significant variation observed in median hospital costs for these admissions. Hospitals varied by nearly six-fold in costs of cardiovascular care in post-KT population. 20% of hospitalizations, as in Table 1, included cardiovascular procedures including cardiac catheterization or cardiac surgery.

Clinical and hospital characteristics were also associated with high cost hospitalizations (HCHs) (Table 4). On univariate analysis, THs were associated with HCH, but after risk-adjustment for both clinical and other hospital characteristics, THs were associated with significantly lower costs than NTHs. Older age and cardiovascular disease burden were associated with significantly higher odds of HCH. Emergent admissions were associated with 46% lower odds of being HCH compared to elective admissions (*p* < 0.001). Cardiovascular procedures were associated with HCH, including diagnostic and therapeutic catheterization, coronary artery bypass grafting, and other cardiac surgery. Higher Medicare payer-mix was a negative predictor for HCH. Higher physician staffing levels was also associated with HCH. The addition of patient and hospital characteristics to TH status led to notable reduction in QIC, suggesting better model fitness.

**Table 4:** Characteristics Associated with High Cost Hospitalizations from Cardiovascular Disease after Kidney Transplantation

Variable	Comparison	Model 1: Transplant Hospital Only				Model 2: Transplant Hospital + Patient Characteristics				Model 3: Transplant Hospital + Patient Characteristics + Hospital Characteristics			
		OR	95% CI		*p*-value	OR	95% CI		*p*-value	OR	95% CI		*p*-value
Transplant hospital	Transplant vs. Non-transplant	1.26	1.04	1.53	0.02	1.46	1.16	1.85	0.002	0.66	0.47	0.94	0.02
Patient-level Characteristics													
Age	≥60 vs. < 60					1.17	0.98	1.40	0.08	1.30	1.05	1.61	0.02
Race	Non-white vs. White					0.89	0.74	1.08	0.25	0.87	0.70	1.10	0.25
Sex	Female vs. Male					0.95	0.80	1.13	0.57	1.05	0.86	1.30	0.62
Type of admission	Emergent/Urgent vs. Elective/Others					0.55	0.43	0.69	< 0.001	0.54	0.40	0.72	< 0.001
Admitted to high transfer in hospital	Yes vs. No					0.97	0.75	1.25	0.81	1.07	0.82	1.40	0.61
Number of CV diagnosis	≥2 vs. 1					1.36	1.16	1.59	< 0.001	1.55	1.25	1.93	< 0.001
Weighted Charlson score	≥2 vs. 0–1					1.08	0.86	1.35	0.51	1.05	0.80	1.39	0.71
Hypertension	Yes vs. No					0.88	0.69	1.12	0.31	0.76	0.56	1.05	0.09
Tobacco abuse	Yes vs. No					0.65	0.42	1.00	0.05	0.68	0.37	1.27	0.23
Dyslipidemia	Yes vs. No					0.79	0.67	0.94	0.006	0.80	0.64	1.01	0.06
Diabetes mellitus	Yes vs. No					0.89	0.76	1.06	0.18	0.89	0.72	1.10	0.29
Invasive CV procedure													
Diagnostic Cardiac catheterization	Yes vs. No					3.65	2.98	4.47	< 0.001	4.51	3.46	5.89	< 0.001
Therapeutic cardiac catheterization	Yes vs. No					3.11	1.79	5.41	< 0.001	3.08	1.57	6.04	0.001
CABG	Yes vs. No					17.91	3.79	84.61	< 0.001	25.48	2.65	244.93	0.005
Valve surgery	Yes vs. No					6.68	1.30	34.31	0.02	13.63	0.66	281.03	0.09
Other cardiac surgery	Yes vs. No					4.06	1.79	9.23	< 0.001	3.88	1.13	13.31	0.03
Hospital-level Characteristics													
Owner, Financial status, Payer Mix													
Hospital total expenses by quartile	2 vs. 1									1.37	0.76	2.49	0.30
	3 vs. 1									1.73	0.89	3.38	0.11
	4 vs. 1									1.40	0.63	3.13	0.41
PPO Hospital	Yes vs. No									1.34	0.80	2.25	0.27
% Medicare discharge	Per 10% increase									0.69	0.60	0.80	< 0.001
Inpatient Capacity													
Hospital unit inpatient days by quartile	2 vs. 1									1.15	0.64	2.06	0.65
	3 vs. 1									0.91	0.48	1.74	0.77
	4 vs. 1									1.03	0.50	2.12	0.93
Staffing Patterns													
Physician FTE/10 beds	Per 1 FTE increase									1.05	1.03	1.07	< 0.001
Nurse FTE/10 beds	Per 1 FTE increase									1.01	1.00	1.03	0.18
Process of Care for Heart Attack													
Aspirin at arrival	Per 5% increase									1.18	0.62	2.24	0.62
Time to ECG	Per 20 min increase									0.99	0.92	1.07	0.85
QIC		6258.81				4193.5				2466.62			

As a sensitivity analysis, we included the 14.2% of hospitalization episodes with inpatient dialysis codes to assess the effect of TH status on mortality and costs when including these hospitalization episodes. Among episodes with dialysis use, there was no differences between THs and NTHs in mortality (THs vs NTHs: 5.9% vs. 7.0%, *p* = 0.51) or high costs admissions (THs vs NTHs: 28.3% vs. 34.5%, *p* = 0.06) on univariate analysis. On multi-variate analysis, Dialysis use did not modify the effect of THs on mortality (no effect of TH status) or high cost care (THs were predictive of lower likelihood of having a high cost episode) (interaction terms for dialysis-transplant hospital status: mortality model *p* = 0.18, high cost care *p* = 0.9). Furthermore, the significant predictors associated with mortality and high cost care did not change, nor was there any notable change in effect size in these models.

## Discussion

We have previously identified two important trends in health care utilization for cardiovascular disease in the transplant population, which fueled our interest in this study. First, utilization of hospital services for cardiovascular disease in the kidney transplant recipients is growing, particularly in non-transplant hospitals and secondly, there was a trend toward higher mortality in these hospitals [5]. By studying a large database of hospitalization episodes and linking it to granular data on hospital characteristics, we were able to design models to identify clinical risk factors and hospital characteristics predictive of adverse clinical and financial outcomes.

An important early finding in the analysis was a concerning trend toward higher inpatient cardiovascular mortality in NTHs. After adjusting for clinical and facility characteristics, THs and NTHs had similar mortality. Clinical factors largely mediated this difference. From the group of facility factors, only teaching status was associated with lower mortality, which was recently also observed by Silber et al. in a Medicare study on MI patients [12]. Predictors of mortality and HCH included age and cardiovascular disease burden (based on the number of coded cardiovascular diagnoses) and utilization of diagnostic cardiac catheterization. Machinicki et al. has previously shown that pre-existing cardiovascular disease burden can reliably predict Medicare mortality and costs in the 3 years following transplantation [20]. Cardiovascular procedures were associated with a lower risk of mortality and higher costs compared to non-procedural admissions, likely related to resources utilized and patient selection in these admissions versus others.

An interesting finding was related to costs of care. While NTHs had longer lengths of stay for the same diagnoses, other significant facility factors were associated with lower costs: higher Medicare payer-mix, lower physician staffing, and TH status. This may imply THs provide better value in managing CVD complications, considering THs and NTHs had similar odds of population-based mortality. Why would this be the case? THs are resource-intense facilities and typically carry significant resources and expertise. This may translate into better value by reducing unnecessary testing or care intensity [21], and could be related to patients being in their “transplant home” where they are a known entity. Practice patterns, in this context, likely differ between THs and NTHs and drive observed differences in HCHs. This finding is novel, and generates a hypothesis that warrants further analysis within specific diagnoses, and potentially with richer clinical data.

This study has direct implications for clinical practice and care models aimed at rescuing post-transplant patients in high-risk cardiovascular scenarios. Prevention of cardiovascular events is key. These events are increasingly recognized and inpatient mortality exceeds 3% [8–11, 14, 20, 22–24]. Risk factor modification should be a central tenet of post-transplant care. Increasing access to preventive cardiology, alterations in immunosuppression, adherence to cardio-protective medication regimens, and application of guideline-based cardiovascular medical therapy may improve outcomes in the post-transplant population [25, 26]. Secondly, our analysis suggests that certain clinical phenotypes are at high risk for mortality – older kidney transplant patients with multiple cardiovascular diagnoses who require invasive interventions. As observed here, transplant patients pursue complex care in all types of facilities – our study indicates that NTHs are associated with reasonable outcomes. This represents a shift from earlier years of clinical transplantation, likely related to greater prevalence of transplant patients in the community and the proliferation of well-resourced hospitals around the country. This shift also warrants the development of formal and informal care networks within communities to manage these patients. Non-transplant providers/facilities take on significant risk with these patients, and transplant providers/hospitals should support them. Formal and informal partnerships underscored by clear inter-facility communication are vital. The development of these networks requires earnest collaboration, and both transplant and non-transplant hospitals have the incentives to do so. Further research evaluating the effectiveness of these networks would be an interesting innovation in studying transplant health services.

This analysis has limitations. Since each record in the data represented a single unlinked hospitalization, the timing of the transplant relative to the cardiovascular event is unknown. Linkage of admissions would have enriched the observations in this analysis, and may have helped elucidate potential interventions for future studies. Administrative data inherently lack clinical granularity which limits our ability to see the true biological effects of documented co-morbidities on in-hospital mortality and costs, such as diabetes and dyslipidemia. The Donabedian model of health care quality prioritizes processes of care, but cardiovascular process measures were not associated with outcomes in this analysis. While these vary between hospitals, they may not be applicable to transplant patient outcomes, or have any effect on outcomes at all [27–29]. All secondary data analyses provide the net effect of specific clinical and hospital-level covariates on outcomes across the entire population, and are subject to the ecological fallacy when evaluating individual outcomes. The effects observed here may also not reflect more recent practice patterns or hospital structural improvements that may affect mortality and costs today.

Adverse outcomes from cardiovascular events impact post-KT survival. Further research is needed to reduce the risk of mortality once an event occurs. Costs related to prevention and cost-effectiveness of event-based care warrant further analysis. These efforts will improve care for transplant patients, optimize rescue in acute settings, reduce post-transplant costs, and extend long-term post-transplant survival.

## Conclusions

Using administrative data, this analysis indicates that transplant and non-transplant hospitals had similar risk-adjusted mortality when managing cardiovascular events in previous kidney transplant recipients. Transplant hospitals were less likely to have high cost episodes of care for these events, which may imply better value in post-transplant cardiovascular care delivery. These data have significant implications for patients, transplant and non-transplant providers, and payers.

## Abbreviations

CART: Classification and Regression Tree
CHF: Congestive heart failure
CT: Computed tomography
CVA: Cerebrovascular accident / stroke
CVD: Cardiovascular disease
GEE: Generalized estimation equations
HCUP: Healthcare Cost and Utilization Project
ICD: 9th International Classification of Disease codes
MI: Myocardial infarction
NIS: Nationwide Inpatient Sample
QIC: Quasi-likelihood under independence criterion
